# Probiotic *Bacillus pumilus* LV149 enhances gut repair, modulates microbiota, and alters transcriptome in DSS-induced colitis mice

**DOI:** 10.3389/fmicb.2024.1507979

**Published:** 2025-01-07

**Authors:** Xinyu Sun, Long Yun, Keming Xie, Renhui Liu, Xinyue Ren, Bokun Zeng, Xudong Cao, Zhi Li, Guihao Zhou, Bang Liu, Luo Peng, Lihong Yuan

**Affiliations:** ^1^School of Life Sciences and Biopharmaceutics, Guangdong Pharmaceutical University, Guangzhou, China; ^2^Huzhou Key Laboratory of Translational Medicine, First Affiliated Hospital of Huzhou University, Huzhou, China; ^3^Medical College of Jiaying University, Jiaying University, Meizhou, China; ^4^School of Sports Medicine, Wuhan Sports University, Wuhan, China; ^5^Department of Chemical and Biological Engineering, University of Ottawa, Ottawa, ON, Canada; ^6^Key Laboratory of Aquacultural Biotechnology Ministry of Education, School of Marine Sciences, Ningbo University, Ningbo, China; ^7^Division of Medicine, University College London, London, United Kingdom; ^8^Key Laboratory of Breeding Biotechnology and Sustainable Aquaculture (CAS), Key Laboratory of Tropical Marine Bio-resources and Ecology, Guangdong Provincial Key Laboratory of Applied Marine Biology, South China Sea Institute of Oceanology, Chinese Academy of Sciences, Guangzhou, China

**Keywords:** *Bacillus pumilus*, ulcerative colitis, gut microbiota, intestinal mucosal barrier, inflammation, transcriptomics

## Abstract

**Purpose:**

Gut microbiota dysbiosis significantly impacts ulcerative colitis (UC) progression and exacerbation. Probiotics show promise in UC management. This study evaluated the effects of different doses of *Bacillus pumilus* LV149, an aquatic-derived probiotic, on gut injury repair in male C57BL/6 mice with dextran sulfate sodium (DSS)-induced ulcerative colitis (UC) and investigated the underlying mechanisms.

**Methods:**

UC was induced by allowing mice free access to a 3% DSS solution for 7 days, with concurrent daily oral gavage of either a low (LV149-L, 1 × 10^8^ CFU/day/mouse) or high (LV149-H, 1 × 10^9^ CFU/day/mouse) dose of LV149. The effects were assessed through physiological parameters, intestinal barrier integrity, inflammation, gut microbiota composition, and transcriptomic changes.

**Results:**

LV149 significantly improved pathological symptoms, including weight loss and disease activity index (DAI), and reduced colon shortening in a dose-dependent manner and inflammatory damage. The intervention also restored gut barrier function by upregulating mucins, goblet cell counts, and tight junction proteins (ZO-1, occludin, and claudin-1) in colonic tissue, along with reducing serum lipopolysaccharide (LPS) levels. Notably, only the LV149-H significantly decreased the expression of pro-inflammatory cytokines TNF-α, IL-1β, and IL-6, while both doses increased the expression of the anti-inflammatory cytokine IL-10 in a dose-dependent in colonic tissue. LV149 further modulated the gut microbiota, increasing beneficial bacteria and reducing pathogenic populations. Transcriptomic analysis indicated that LV149-L may exert gut repair effects via the IL-17 signaling pathway, whereas LV149-H appears to act through the JAK-STAT signaling pathway.

**Conclusion:**

This study demonstrated that LV149, particularly at a higher dose, effectively mitigated DSS-induced colonic injury by modulating gut microbiota, enhancing gut barrier integrity, and reducing inflammation. The dose-dependent effects underscored LV149-H’s potential as a therapeutic agent for UC due to its stronger anti-inflammatory properties and gut-protective effects.

## 1 Introduction

Ulcerative colitis (UC) is a chronic, relapsing inflammatory bowel disease (IBD) characterized by persistent mucosal inflammation that can extend from the distal to the proximal colon and may eventually involve the entire colon (Segal et al., 2021). In North America and Northern Europe, UC has an incidence rate ranging from 9 to 20 cases per 100,000 people, with a prevalence rate between 156 and 291 cases per 100,000 individuals (Feng et al., 2017; Le Berre et al., 2023). Clinical management of UC primarily relies on surgical and pharmacological treatments. However, surgical interventions are often associated with higher postoperative mortality (Singh et al., 2015), while long-term pharmacotherapy can lead to adverse effects (Ordás et al., 2012). Although the precise etiology of UC remains unclear, several factors have been identified as contributing to its development and progression, including epithelial barrier dysfunction, gut microbiota dysbiosis, and chronic inflammation (Allam-Ndoul et al., 2020; Eastaff-Leung et al., 2010; Kumar and Kumar, 2022; Mehandru and Colombel, 2021).

The intestinal barrier plays a critical role in protecting the body from pathogens and toxins while selectively allowing nutrients to pass through (Camilleri et al., 2012). In UC patients and animal models, damage to the intestinal barrier leads to an increased influx of antigens and bacterial components into the intestinal lumen, which triggers immune responses and exacerbates inflammation (Allam-Ndoul et al., 2020; Mehandru and Colombel, 2021; Yu, 2018). Furthermore, inflammatory mediators can impair the secretion of tight junction proteins, widening the intercellular gaps and increasing intestinal permeability (Al-Sadi et al., 2010). This feedback loop contributes to further barrier dysfunction and tissue damage. Studies have shown that higher levels of tight junction protein expression correlate with better prognosis in UC patients (Tan et al., 2019). As such, restoring the intestinal barrier and maintaining its function represent key therapeutic targets for UC.

Dysbiosis, or an imbalance in gut microbiota, has been linked to mucosal injury, immune system dysfunction, and chronic colonic inflammation (Adak and Khan, 2019; Eslami et al., 2019; Guo et al., 2020; Yuan et al., 2021). Both clinical and animal studies of UC have revealed a reduction in beneficial bacteria, such as *Bifidobacterium*, *Lactobacillus*, and *Clostridia*, and an increase in potentially harmful bacteria, such as *Enterococci* and *Escherichia* (Chen et al., 2022; Cui et al., 2021; Li Y. et al., 2022; Sanders et al., 2019; Wang X. et al., 2022). Moreover, chronic inflammation may create a selective environment that favors the survival of specific taxa, such as *Proteobacteria*, under oxidative stress conditions (Wang et al., 2022a; Winter and Bäumler, 2014). Therefore, addressing microbial imbalances represents a promising strategy for UC therapy. Restoring microbial homeostasis and improving gut barrier function through probiotics—such as *Pediococcus pentosaceus* (Dong et al., 2022), *Akkermansia muciniphila* (Qu S. et al., 2021), *Lactobacillus acidophilus* (Wang et al., 2018), *Bifidobacterium infantis* (Han et al., 2021), *Clostridium butyricum* (Stoeva et al., 2021), and *Faecalibacterium prausnitzii* (Miquel et al., 2013) has shown promise in alleviating UC symptoms.

Interestingly, *Bacillus pumilus*, a probiotic widely used in agriculture, animal husbandry, and aquaculture, has demonstrated multiple beneficial properties, including promoting plant growth, enhancing crop yield, and improving animal intestinal health (Bilal et al., 2021; Dobrzyński et al., 2022; Hill et al., 2009; Liu et al., 2023; Zeng et al., 2022; Zhang et al., 2020; Zhang et al., 2021). A strain of *Bacillus pumilus* LV149 was previously isolated from the gut mucosa of healthy *Litopenaeus vannamei* and shown to exhibit significant extracellular protease, lipase, and amylase activities, while effectively inhibiting *Vibrio parahaemolyticus* infection without causing hemolysis (CN201210029204.9). Given these properties, *Bacillus pumilus* LV149 may hold potential as a probiotic for human gastrointestinal health. However, to date, no studies have explored its role in gut injury repair.

Therefore, this study aims to evaluate the gut injury repair effects of different doses of *Bacillus pumilus* LV149 in DSS-induced UC model mouse. Additionally, we investigate the underlying mechanisms of repair through transcriptomic analysis and gut microbiota profiling.

## 2 Materials and methods

### 2.1 Preparation of LV149 suspensions

The *Bacillus pumilus* LV149 bacterial strain (deposited at CCTCC, No. M 2011411, GenBank No. JQ036323.1) was inoculated into LB solid medium and cultured overnight at 37°C and subcultued every 12 h for 3 consecutive times. Subsequently, a single colony was selected and transferred to LB liquid medium and cultured overnight at 37°C with shaking at 150 rpm. The culture was inoculated at a 3% inoculation ratio into fresh LB liquid medium and incubated at 37°C with shaking at 150 rpm. Cultivation continued until the optical density at 600 nm (OD600) reached approximately 0.5, corresponding to a concentration of the LV149 suspension at approximately 1 × 10^8^ CFU/mL. The LV149 were harvested by centrifugation at 6,500 g for 5 min at 4°C, washed twice with ice-cold physiological saline solution (PSS, 0.9% NaCl), and resuspended in PSS to achieve bacterial concentrations of 1 × 10^9^ CFU/mL and 1 × 10^8^ CFU/mL. Finally, the resuspended LV149 bacterial suspension was administered to mice via gavage within 2 h.

### 2.2 Experimental design and mouse model for UC

In this study, a total of 40 eight-week-old SPF C57BL/6 male mice (20–22) g were purchased from the Guangdong Provincial Medical Experimental Animal Center and were raised in an SPF-level environment at the Animal Experimental Center of Guangdong Pharmaceutical University. Throughout the experiment, the mice were maintained in a room with a controlled temperature of 23 ± 2°C and a relative humidity of 50 ± 5% under a 12-h light-dark cycle. The mice were provided with SPF-grade mouse feed and bedding. All animal studies received approval from the Animal Protection and Use Committee at Guangdong Pharmaceutical University (Approval No. gdpulacspf 2022325).

**Figure 1:**
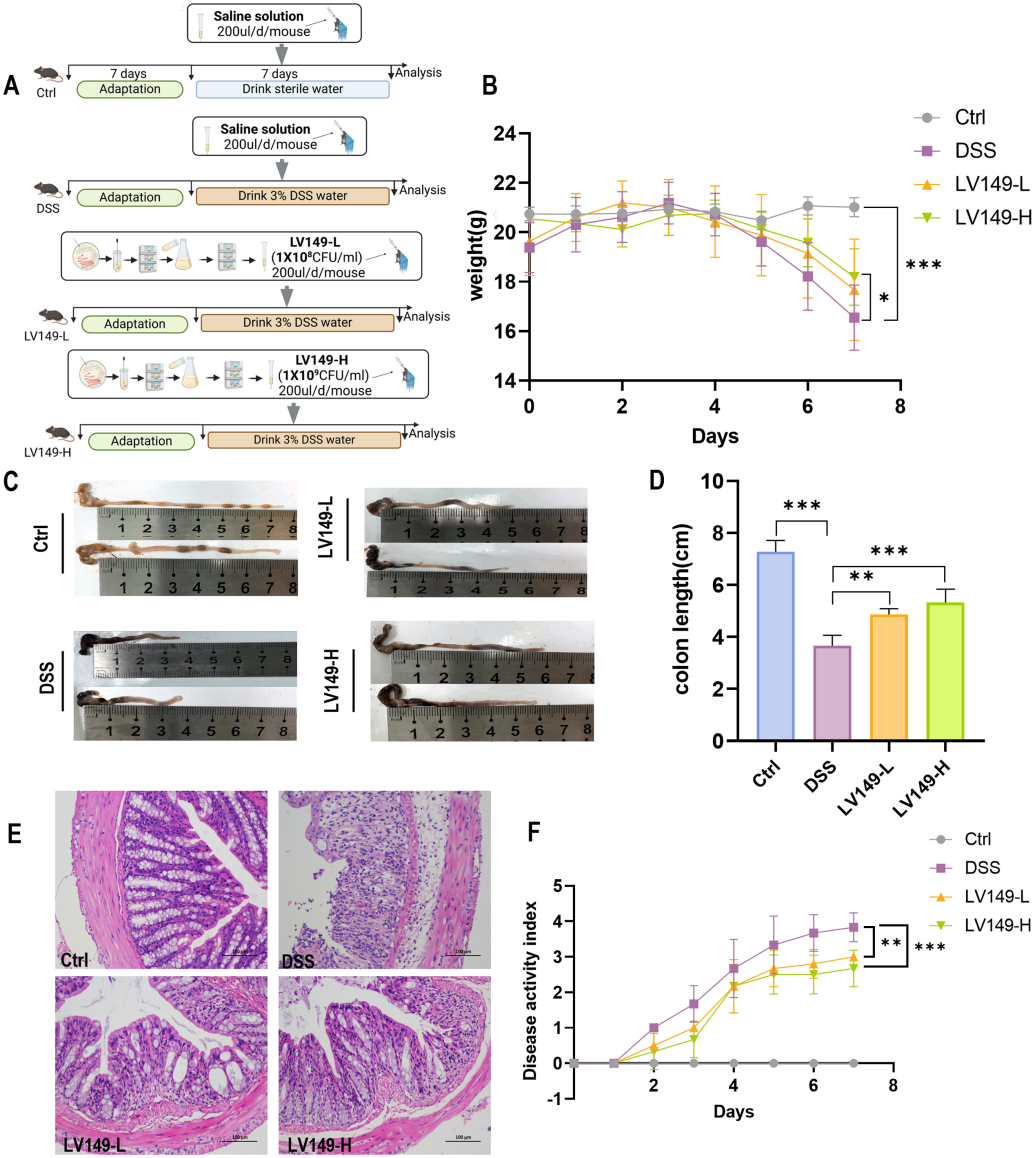
LV149 improves the pathological symptoms of DSS-induced UC model mice. **(A)** Experimental design. **(B)** Daily changes in body weight of mice during the experiment (*n* = 10). **(C,D)** Images of mice colon length and statistical analysis from each group (*n* = 5). **(E)** H&E staining of colon tissue sections from each group (scale bars, 100 μm, 200×). **(F)** Disease activity index of different groups (*n* = 6). Data are expressed as mean ± standard deviation. ^*^p < 0.05, ^**^p < 0.01, and ^***^p < 0.001 (one-way ANOVA).

Prior to the experiments, the mice were acclimated for 1 week. The mice were then randomly divided into four groups, with each group containing 10 mice. The groups consisted of control group (Ctrl), DSS model group (DSS), low concentration group (LV149-L) and high concentration group (LV149-H). Specifically, mice in the Ctrl group were provided with normal drinking water and mouse feed; Mice in the DSS and LV149 groups were administered 3% (wt/vol) dextran sulfate sodium (DSS) via drinking water for 7 days. In addition to the DSS administration, mice in the DSS groups were also subjected to intragastric administration of PSS (200 μL/mouse/day). The LV149-L group was given a bacterial suspension (1 × 10^8^ CFU/mouse/day) and the LV149-H group a higher dosage (1 × 10^9^ CFU/mouse/day) through intragastric administration. Mice were euthanized at the end of the experiment by cervical dislocation. The experimental protocol is illustrated in Figure 1A.

### 2.3 Sample collection

In accordance with the experimental procedure, the colonic tissue from the mice was removed to measure its length. After rinsing the colon tissue with pre-chilled phosphate-buffered saline (PBS, pH 7.4), it was promptly divided into two parts: one segment was fixed using a 4% paraformaldehyde solution, while the other was frozen at −80°C. In addition, serum from the experimental animals was collected via eyeball extraction. The freshly collected blood was placed in an anticoagulant tube and then centrifuged at 4°C at a speed of 4,000 rpm for 15 min. Finally, the serum was stored at −80°C until it was ready for analysis.

### 2.4 Histopathological evaluation

The distal colonic tissues were fixed in a 4% paraformaldehyde solution, embedded in paraffin, and sectioned into 5-μm thick slices. For histopathological analysis, the sections were stained with hematoxylin and eosin (H&E) for general tissue structure assessment or with Alcian blue to visualize mucopolysaccharides. The stained samples were then observed and documented using a Nikon Eclipse E100 microscope (Nikon, Tokyo, Japan).

### 2.5 ELISA analysis

Colonic inflammatory cytokines, including interleukin-1β (IL-1β; Catalog No. ED-20174), interleukin-6 (IL-6; Catalog No. ED-20188), tumor necrosis factor-α (TNF-α; Catalog No. ED-20852), and interleukin-10 (IL-10; Catalog No. ED-20162), were quantified from colonic tissue samples using enzyme-linked immunosorbent assay (ELISA) kits. Serum lipopolysaccharides (LPS; Catalog No. ED-20839) were quantified separately. All measurements were performed using ELISA kits (Xiamen Lunchangshuo Biotechnology Co., Ltd., Xiamen, China) according to the manufacturer’s instructions.

### 2.6 RNA extraction and quantitative real-time PCR analysis

Total mRNA was extracted from colonic tissue samples using TRIzol reagent (Invitrogen, Carlsbad, CA, United States), following the manufacturer’s protocol. Complementary DNA (cDNA) synthesis was performed using the PrimeScript^™^ RT reagent kit with gDNA Eraser (Takara, Catalog No. RR047A, Japan) to remove genomic DNA contamination. Quantitative real-time PCR (qRT-PCR) was conducted with the ChamQ SYBR qPCR Master Mix (Vazyme Biotech Co., Ltd., Catalog No. Q321, Nanjing, China). Gene-specific primers used in this study are provided in Supplementary Table S1. Gene expression levels were normalized to the GAPDH gene, and relative expression was calculated using the $2^{-\Delta \Delta C_{T}}$ method (Wang et al., 2022b).

### 2.7 Immunofluorescence analysis

Immunofluorescence (IF) staining was performed to assess tight junction proteins (ZO-1, occludin, and claudin-1). Colonic tissue sections (5 μm) were deparaffinized in xylene, rehydrated through graded ethanol, and subjected to antigen retrieval in citrate buffer (pH 6.0) at 95°C for 30 min.

After antigen retrieval, sections were blocked with 5% bovine serum albumin (BSA) in PBS for 1 h at room temperature. Primary antibodies were applied overnight at 4°C: rabbit anti-mouse occludin (1:500, Catalog No. GB111401; Servicebio, Wuhan, China), rabbit anti-mouse ZO-1 (1:500, Catalog No. GB111402; Servicebio, Wuhan, China), and rabbit anti-mouse claudin-1 (1:500, Catalog No. GB11032; Servicebio, Wuhan, China).

Following incubation with primary antibodies, sections were washed three times in PBS. The corresponding Cy3-conjugated goat anti-rabbit IgG (H + L) secondary antibody (1:300, Catalog No. GB21303; Servicebio, Wuhan, China) was applied for 1 h at room temperature. After secondary antibody incubation, sections were washed and stained with DAPI (Catalog No. GB1012; Servicebio, Wuhan, China) for nuclear counterstaining. Fluorescence was captured using a Nikon Eclipse C1 microscope (Nikon, Japan). Fluorescence intensity was analyzed using ImageJ software (Version 1.54), with relative protein expression quantified by measuring integrated optical density (IOD) normalized to tissue area (Wang et al., 2022a).

### 2.8 Gut microbiota analysis

Fecal samples from mice were collected for total bacterial DNA extraction using the E.Z.N.A.^®^ Stool DNA Kit (Omega Bio-tek, Norcross, GA), following the manufacturer’s instructions. The V1–V9 regions of the 16S rRNA gene were amplified by PCR with primers 27F (5′-AGRGTTYGATYMTGGCTCAG-3′) and 1492R (5′-RGYTACCTTGTTACGACTT-3′). SMRTbell libraries were purified and sequenced on PacBio Sequel II 8M cells using Sequencing Kit 2.0 chemistry, provided by Shanghai Biozeron Biotechnology Co., Ltd. (Shanghai, China), as described in a previous study (Shen et al., 2023).

OTUs were clustered at a 98.65% similarity threshold using UPARSE (version 7.1; http://drive5.com/uparse/). Phylogenetic classification of the 16S rRNA sequences was performed using the RDP Classifier^1^ against the Silva (SSU132) 16S rRNA database. Alpha diversity was assessed at the OTU level using Mothur (version 1.21.1; https://mothur.org/wiki/mothur_v.1.21.1), while beta diversity was analyzed using UniFrac. Principal coordinates analysis (PCoA) based on Bray-Curtis distances was performed using the Vegan 2.0 package from R-forge.^2^

Linear discriminant analysis effect size (LEfSe) was performed to identify biomarkers among colonic bacteria, incorporating the Kruskal–Wallis sum-rank test to assess differences across groups, followed by linear discriminant analysis (LDA) to determine the effect size of differentially abundant taxa. Functional changes in the microbiota were predicted using the PICRUSt program, based on the Kyoto Encyclopedia of Genes and Genomes (KEGG) database. OTU data were processed to generate BIOM files compatible with PICRUSt v1.1.09 using the make.biom script in Mothur. OTU abundances were linked to Greengenes OTU IDs for functional inference (Lu et al., 2024).

### 2.9 Transcriptomic analysis

Total RNA was extracted from the colon tissue using a TRIzol^®^ Reagent according the manufacturer’s instructions (Invitrogen) and genomic DNA (TaKara). RNA-seq transcriptome libraries were prepared using the TruSeq^™^ RNA Sample Preparation Kit (Illumina, San Diego, CA). Paired-end libraries were sequenced by Illumina NovaSeq 6000 sequencing (150 bp*2, Shanghai Biozeron Co., Ltd.). The raw reads were spliced to generate clean reads, which were then processed further to remove Illumina adapters and eliminate low-quality reads. Then clean reads were separately aligned to reference genome with orientation mode using hisat2^3^ software to obtain the mapped data (reads) for subsequent analysis.

Differentially expressed genes between samples were identified by calculating gene expression levels using the FPKM method. Genes were considered differentially expressed genes (DEGs) with significance levels of *p* < 0.05 and |log2FC| ≥1. To explore the functions of DEGs, GO enrichment and KEGG pathway analyses were conducted using Goatools^4^ and KOBAS.^5^

### 2.10 Statistical analysis

Statistical analysis was performed using one-way analysis of variance (ANOVA), followed by Tukey’s multiple comparison test. A *p*-value of less than 0.05 was considered statistically significant. The bacterial community function was predicted using the Wilcoxon rank-sum test. To assess the correlation between gut microbiota and DEGs influenced by LV149, Pearson’s correlation coefficient was calculated.

## 3 Results

### 3.1 *Bacillus pumilus* LV149 ameliorated the pathological symptoms in DSS-induced UC model mice

Compared with the Ctrl group, mice in the DSS group exhibited a significant decrease in body weight and shorter colon length by day 7 of the experiment (Figures 1B–D). Additionally, DAI was significantly elevated in the DSS group (Figure 1F). Histological analysis through H&E staining revealed noticeable infiltration of inflammatory cells, mucosal damage, and crypt loss in the colonic tissue of the DSS group, confirming the successful induction of the UC model by DSS (Figure 1E). In contrast, these pathological changes were significantly ameliorated following oral gavage of LV149 (Figures 1B–F). These findings indicated that LV149 effectively alleviated the pathological symptoms in DSS-induced UC model mice.

### 3.2 *Bacillus pumilus* LV149 ameliorated intestinal barrier damage in DSS-induced UC model mice

**Figure 2:**
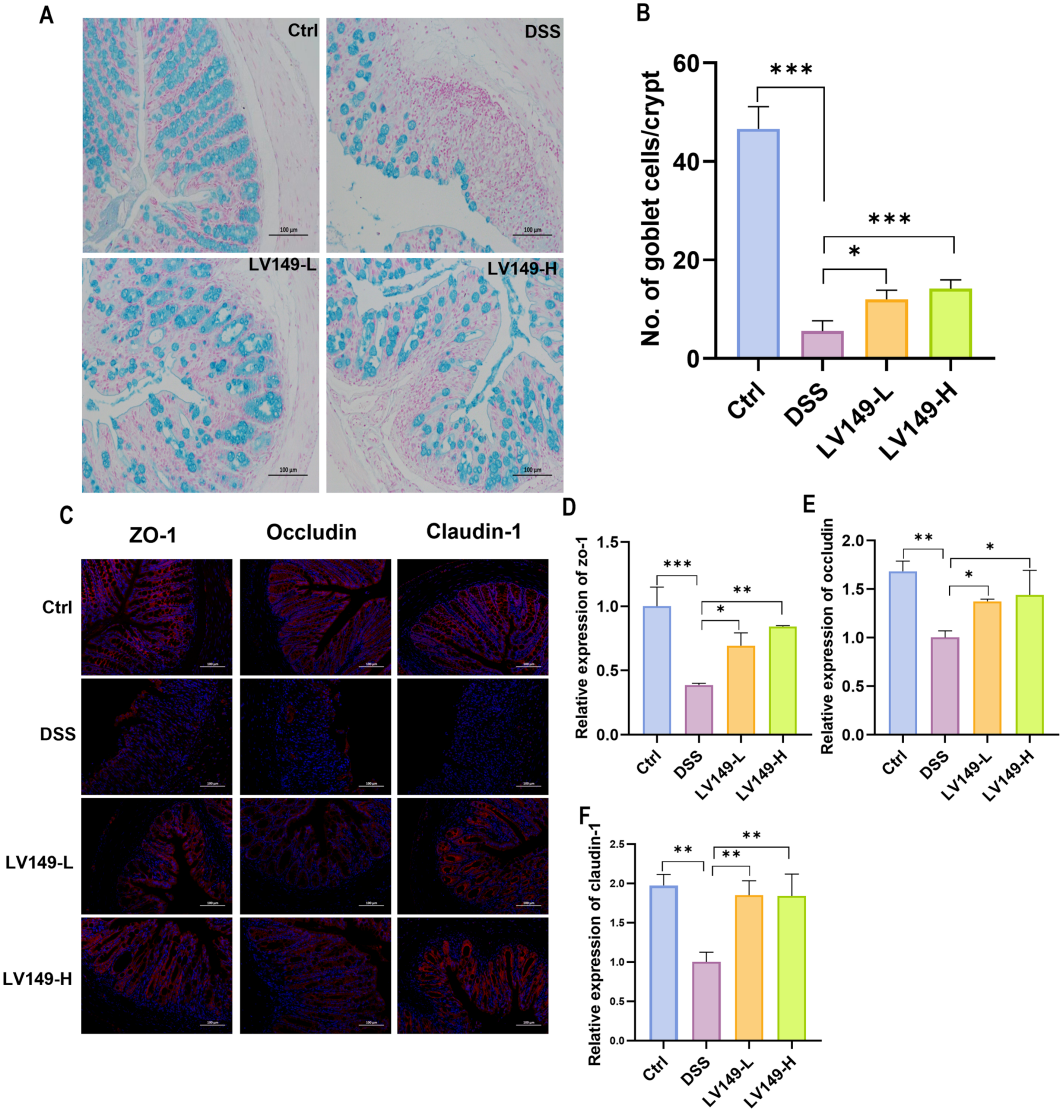
LV149 improves intestinal barrier damage in DSS-induced UC model mice. **(A)** Colon tissue section stained with Alcian blue (scale bars, 100 μm, 200×). **(B)** Goblet cell number in each crypt (*n* = 5). **(C)** Immunofluorescence analysis of ZO-1, occludin, and claudin-1 in colon tissue sections (scale bars, 100 μm, 200×). Relative quantification of ZO-1 **(D)**, occludin **(E)** and claudin-1 **(F)** using ImageJ fluorescence (*n* = 3). Data are expressed as mean ± standard deviation. ^*^p < 0.05, ^**^p < 0.01, and ^***^p < 0.001 (one-way ANOVA).

As shown in Figure 2A, compared with the Ctrl group, DSS group displayed a decrease in the number of goblet cells in Alcian blue-stained colonic tissues. This reduction indicated a decrease in mucin production, which is essential for the mucus barrier in the colon. Furthermore, Alcian blue-stained images revealed pathological damage, inflammatory cell infiltration, and edema in the mucosal layer of the DSS group, highlighting the severity of colon tissue damage. However, following oral gavage of LV149, the number of goblet cells significantly increased in a dose-dependent manner (Figure 2B).

To further investigate the impact of LV149 on the integrity of the intestinal barrier, the expression levels of tight junction (TJ) proteins, including ZO-1, occludin, and claudin-1, were measured at the protein level. In the Ctrl group, the expression of these three tight junction proteins was abundant and evenly distributed (Figure 2C). In contrast, in the DSS group, the expression of all three TJ proteins was significantly reduced. However, in both LV149-H and LV149-L groups, the expression of these proteins significantly increased and approached levels seen in the Ctrl group, with a more uniform distribution. The relative expression levels of the TJ proteins were further quantified and are shown in Figures 2D–F. It was evident that in comparison with the Ctrl group, the relative expression levels of ZO-1, occludin, and claudin-1 in the DSS-induced UC model mice were significantly decreased. In contrast, the LV149 groups showed a significant dose-dependent increase in the relative expression of these tight junction proteins.

These results collectively suggested that LV149 effectively repaired the integrity of the intestinal barrier and may enhance intestinal barrier function.

### 3.3 *Bacillus pumilus* LV149 reduced colonic inflammation in DSS-induced UC model mice

**Figure 3:**
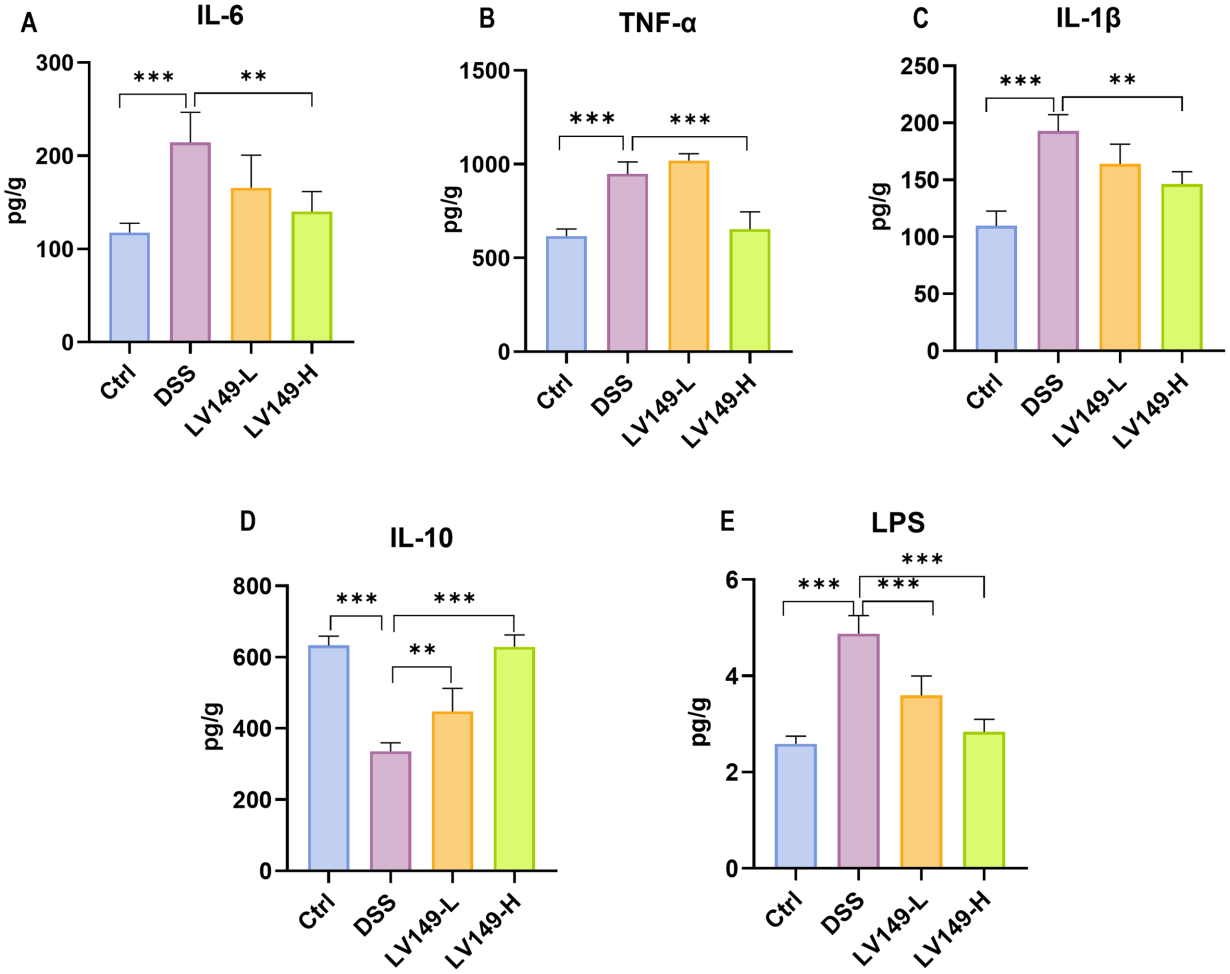
LV149 ameliorates DSS-induced colonic inflammation in DSS-induced UC model mice. **(A–C)** The concentration of colonic pro-inflammatory cytokines IL-6, TNF-α, and IL-1β (*n* = 3–5). **(D)** The concentration of colonic anti-inflammatory cytokine IL-10 (*n* = 3–5). **(E)** The concentration of serum LPS (*n* = 7). ^*^p < 0.05, ^**^p < 0.01, and ^***^p < 0.001 (one-way ANOVA).

As shown in Figures 3A–C, the levels of pro-inflammatory cytokines IL-1β, IL-6, and TNF-α in the Ctrl group were significantly lower than those in the DSS group. Compared with the DSS group, the LV149-H group exhibited a significant reduction in the levels of all three pro-inflammatory cytokines. In contrast, the LV149-L group demonstrated only a trend toward decreased IL-1β and IL-6 levels. Regarding the anti-inflammatory cytokine IL-10, the colonic tissues of DSS group showed a marked reduction in IL-10 levels compared with the Ctrl group. However, LV149 significantly enhanced IL-10 expression in the colonic tissues in a dose-dependent manner, as compared with the DSS group (Figure 3D). Additionally, the levels of LPS in the LV149 groups were significantly reduced in a dose-dependent manner, as shown in Figure 3E, and were lower than those observed in the DSS group. These results suggested that LV149 effectively suppresses the inflammatory response in the colonic tissues of DSS-induced UC model mice.

### 3.4 *Bacillus pumilus* LV149 improved gut microbiota dysbiosis in DSS-induced UC model mice

**Figure 4:**
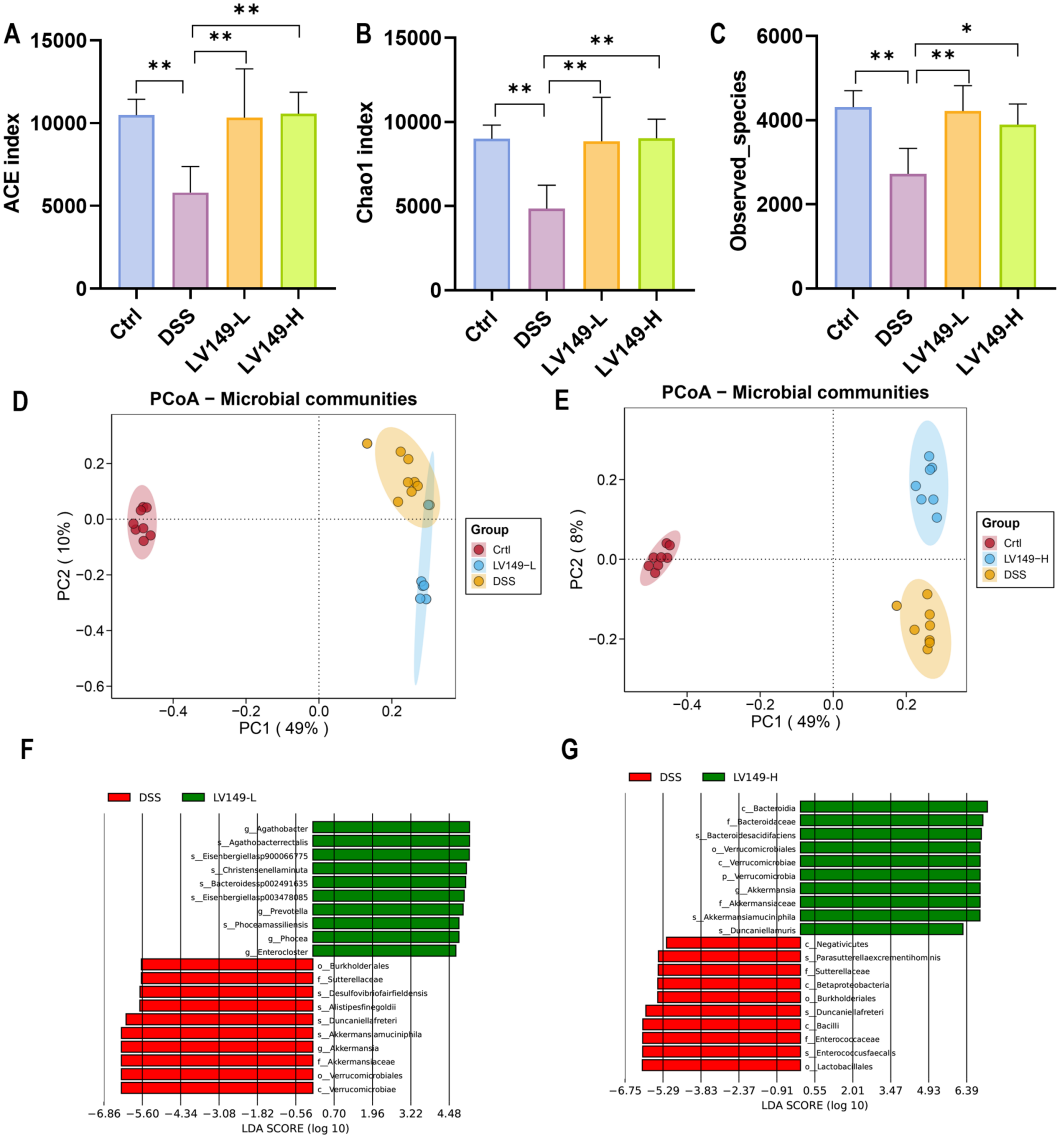
LV149 modulates gut microbiota composition in DSS-induced UC model mice. **(A–C)** Alpha diversity indices (ACE, Chao1, and observed species) indicating community richness across groups (*n* = 5). **(D,E)** PCoA analysis based on Bray–Curtis distances of gut microbiota, with each point representing an individual biological replicate (*n* = 7–8). **(F,G)** Taxonomic cladogram from LEfSe analysis comparing DSS with LV149-L and LV149-H groups. Data are presented as mean ± standard deviation. ^*^p < 0.05, ^**^p < 0.01, and ^***^p < 0.001 (one-way ANOVA).

The study conducted 16S rRNA gene sequencing of the cecal contents from UC mice to assess the effect of LV149 on the gut microbiota. The rarefaction and rank abundance curves showed a saturation trend, indicating that additional sequencing depth was unnecessary and that the majority of bacterial species had likely been identified (Supplementary Figure S1). Venn diagram analysis revealed that 1,425 species were shared across the samples (Supplementary Figure S2). The ACE, Chao1, and Observed species indices, which reflect the diversity of the intestinal microbiota, showed that the DSS group had significantly lower species richness than the Ctrl group. However, the LV149-L and LV149-H groups showed a significant reversal of this trend, with microbial diversity approaching that of the Ctrl group (Figures 4A–C). These results suggested that LV149 played a positive role in restoring microbial diversity in DSS-induced UC model mice.

Principal component analysis (PCA) (Supplementary Figures S3A,B) and principal coordinate analysis (PCoA) (Figures 4D,E) were used to compare sample similarity across different groups. The results showed that samples within the Ctrl, DSS, LV149-L, and LV149-H groups clustered closely, indicating similar gut microbiota profiles within each group. In contrast, samples from the DSS group were significantly different from the Ctrl group along both PC1 and PC2 axes, highlighting distinct microbiota profiles in DSS-induced UC model mice. The LV149-L and LV149-H groups showed partial similarity to the DSS group along PC1, but were more distantly separated along PC2, indicating some differences in microbiota composition between these groups and the DSS group. These findings suggested that LV149 modulated the gut microbiota community structure in DSS-induced UC model mice.

**Figure 5:**
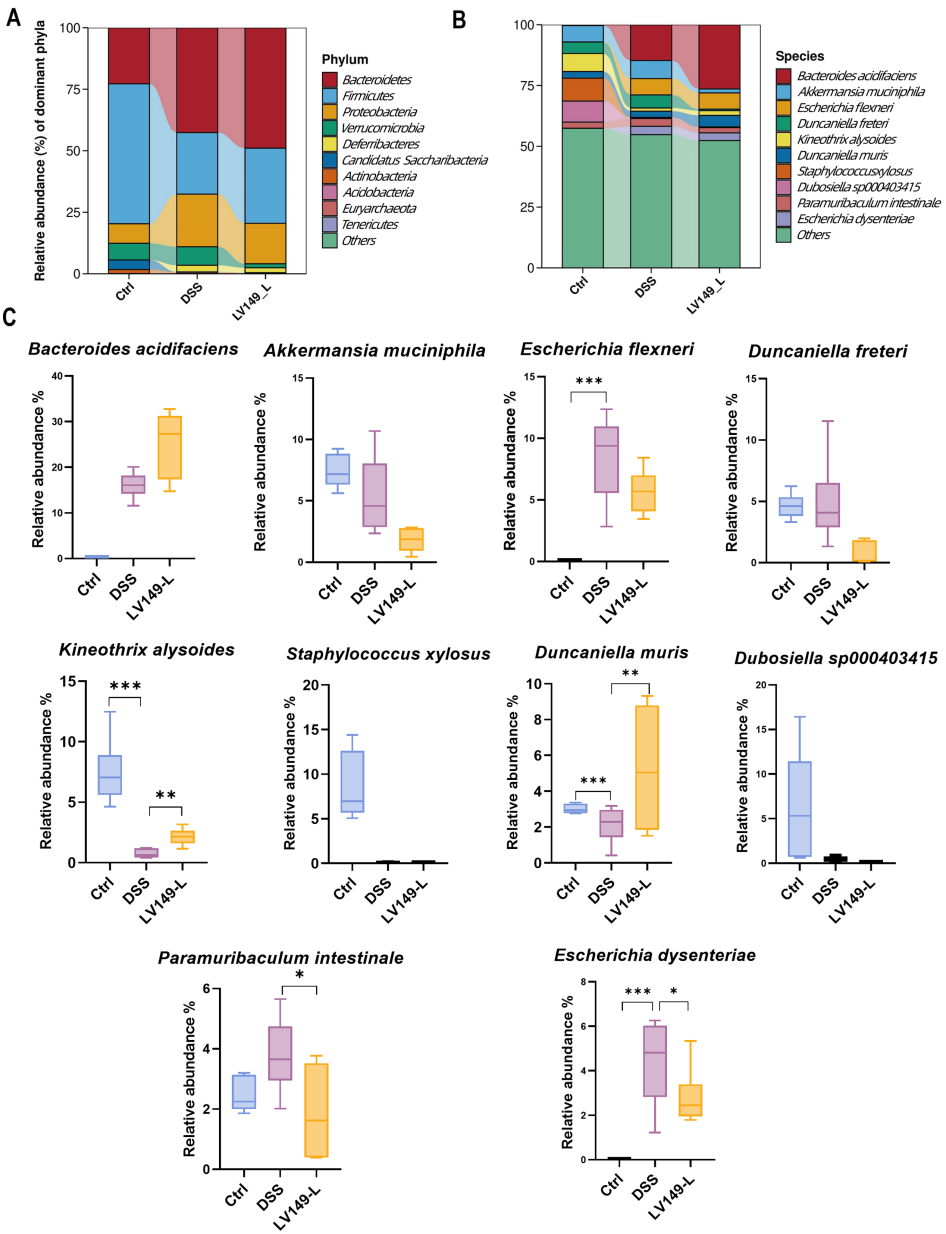
LV149-L modulates microbiota composition in DSS-induced UC model mice. **(A,B)** Relative abundance of the top 10 taxa in the microbial community at the phylum and species levels. **(C)** Changes in the abundance of the top 10 species across different groups (*n* = 6). Data are presented as mean ± standard deviation. ^*^p < 0.05, ^**^p < 0.01, and ^***^p < 0.001 (one-way ANOVA).

**Figure 6:**
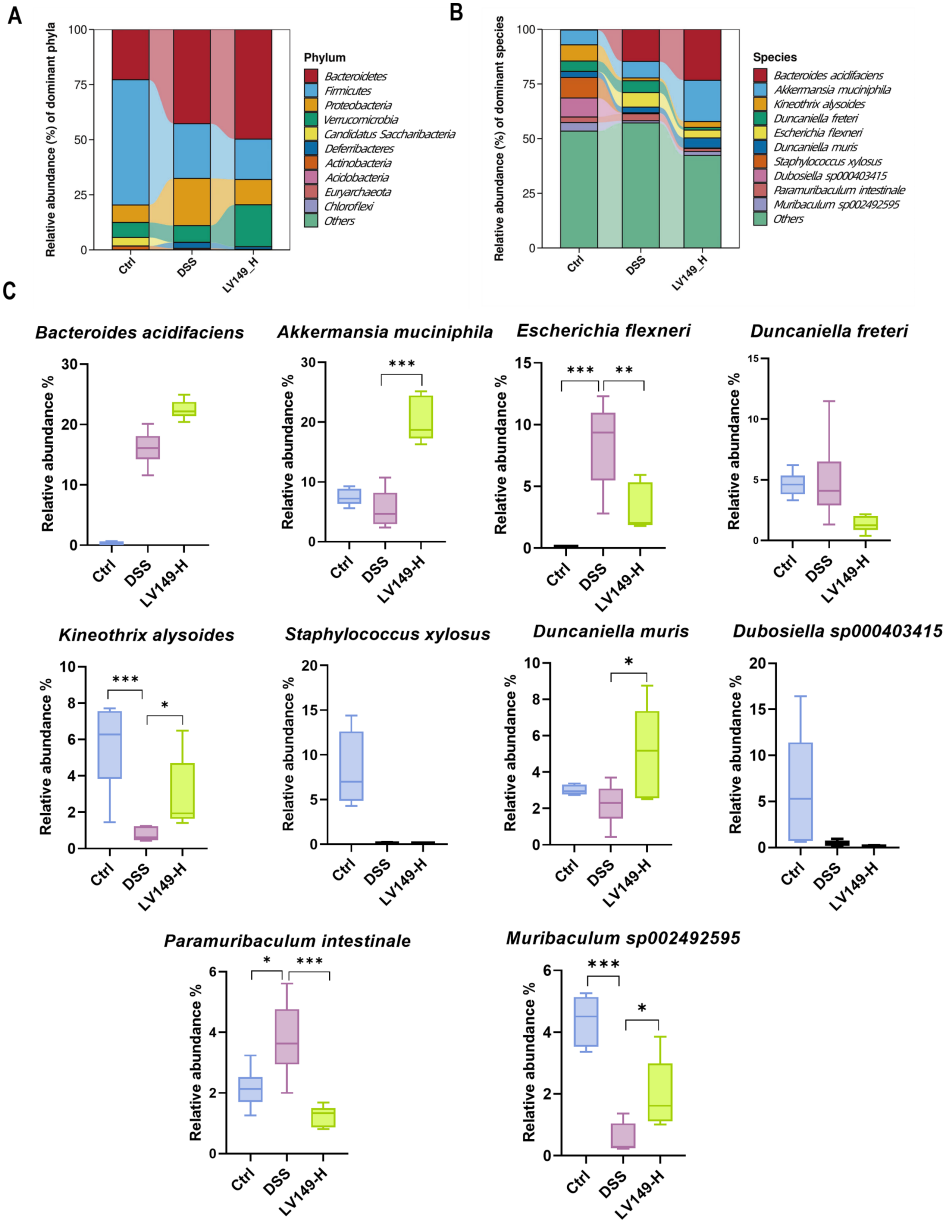
LV149-H modulates microbiota composition in DSS-induced UC model mice. **(A,B)** Relative abundance of the top 10 taxa in the microbial community at the phylum and species levels. **(C)** Changes in the abundance of the top 10 species across different groups (*n* = 6). Data are presented as mean ± standard deviation. ^*^p < 0.05, ^**^p < 0.01, and ^***^p < 0.001 (one-way ANOVA).

Further investigation using Linear discriminant analysis Effect Size (LEfSe) analysis revealed significant bacterial taxa differences between groups (Figures 4F,G). Compared with the DSS group, the LV149-L and LV149-H groups had 4 significant bacterial genera (*Agathobacter*, *Prevotella*, *Phocea*, and *Enterocloster*) and 1 significant bacterial genus (*Akkermansia*), respectively. These taxa may play an important role in the observed changes in microbial community structure within the LV149 groups. At the phylum level, *Bacteroidetes* was the predominant phylum among the top 10 taxa, followed by *Firmicutes* and *Proteobacteria*. The DSS group showed increased relative abundances of *Bacteroidetes*, *Proteobacteria*, and *Deferribacteres*, while *Firmicutes* was decreased compared with the Ctrl group. After LV149-L and LV149-H intervention, these alterations were partially restored. Specifically, LV149-L and LV149-H reduced the relative abundances of *Proteobacteria* and *Deferribacteres* to levels similar to those of the Ctrl group. Notably, only LV149-L increased the relative abundance of *Firmicutes*, restoring it to levels comparable to the Ctrl group (Figures 5A, 6A).

At the species level, LV149-L significantly increased the growth of *Kineothrix alysoides* and *Duncaniella muri*s and decreased the growth of *Paramuribaculum intestinale* and *Escherichia dysenteriae* to levels similar to the Ctrl group (Figures 5B,C). Similarly, LV149-H significantly upregulated the growth of *Akkermansia muciniphila*, *Kineothrix alysoides*, *Duncaniella muris*, and *Muribaculum sp00292595*, while significantly downregulating the growth of *Escherichia flexneri* and *Paramuribaculum intestinale*, all to levels similar to the Ctrl group (Figures 6B,C).

**Figure 7:**
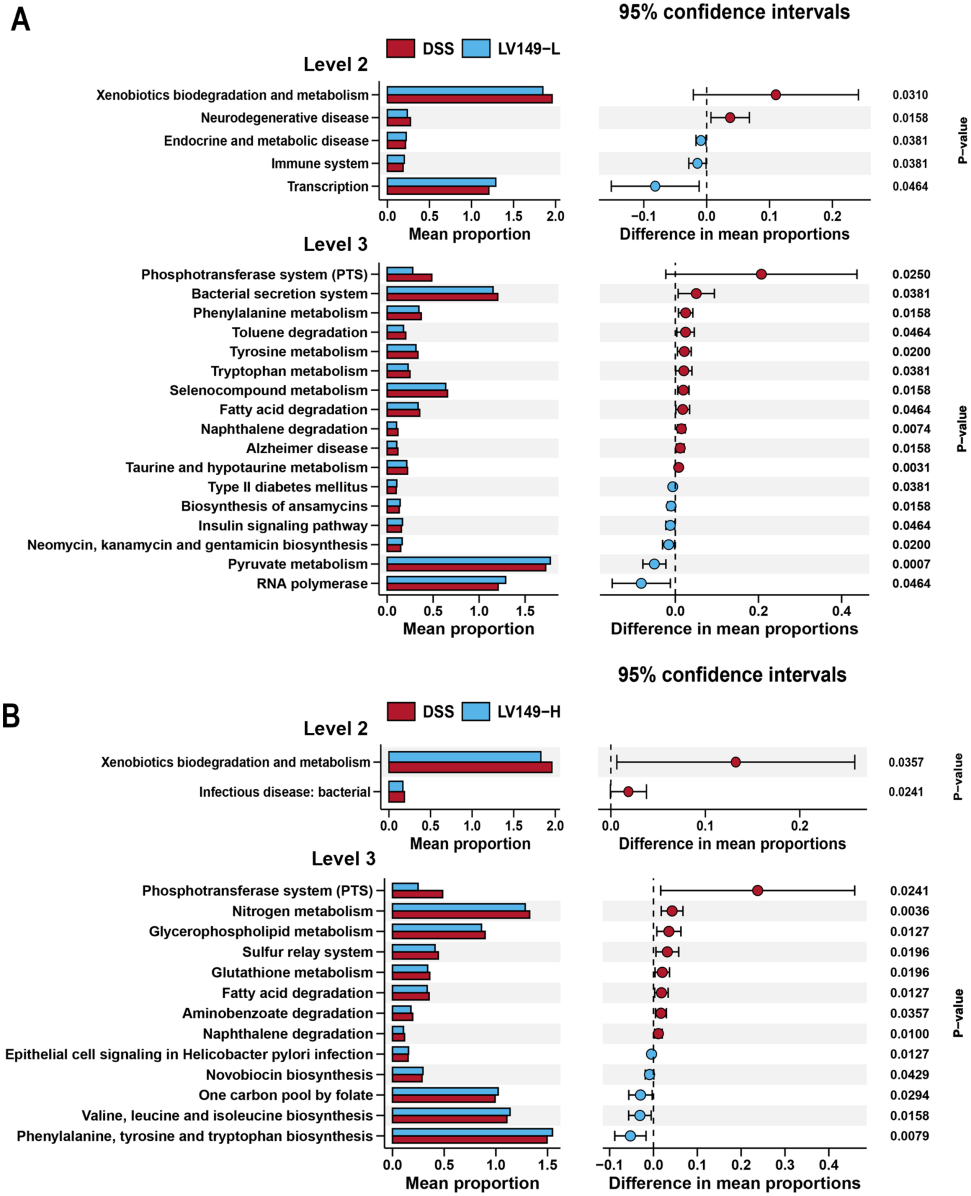
KEGG functional predictions and analysis of LV149 effects using PICRUSt2. **(A)** Comparison between LV149-L and DSS groups. **(B)** Comparison between LV149-H and DSS groups.

To examine the functional profiles of the intestinal microbiota, PICRUSt analysis based on KEGG pathways was performed. As shown in Figure 7A, when comparing the functional profiles of the LV149-L group to the DSS group, the DSS group showed a significant increase in functional modules at level 2, including xenobiotics biodegradation and metabolism, as well as neurodegenerative disease. At level 3, the DSS group exhibited an increase in functional modules such as phosphotransferase system, bacterial secretion system, phenylalanine metabolism, toluene degradation, tyrosine metabolism, tryptophan metabolism, selenocompound metabolism, fatty acid degradation, naphthalene degradation, Alzheimer disease, taurine and hypotaurine metabolism.

As shown in Figure 7B, when comparing the functional profiles of the LV149-H group with those of the DSS group, the DSS group showed an increase in functional modules at level 2, including xenobiotics biodegradation and metabolism, and infectious diseases (bacterial). At level 3, the DSS group showed significant increases in functional modules such as phosphotransferase system, nitrogen metabolism, glycerophospholipid metabolism, sulfur relay system, glutathione metabolism, fatty acid degradation, aminobenzoate degradation, and naphthalene degradation.

### 3.5 *Bacillus pumilus* LV149 altered transcriptional profiles in DSS-induced UC model mice

**Figure 8:**
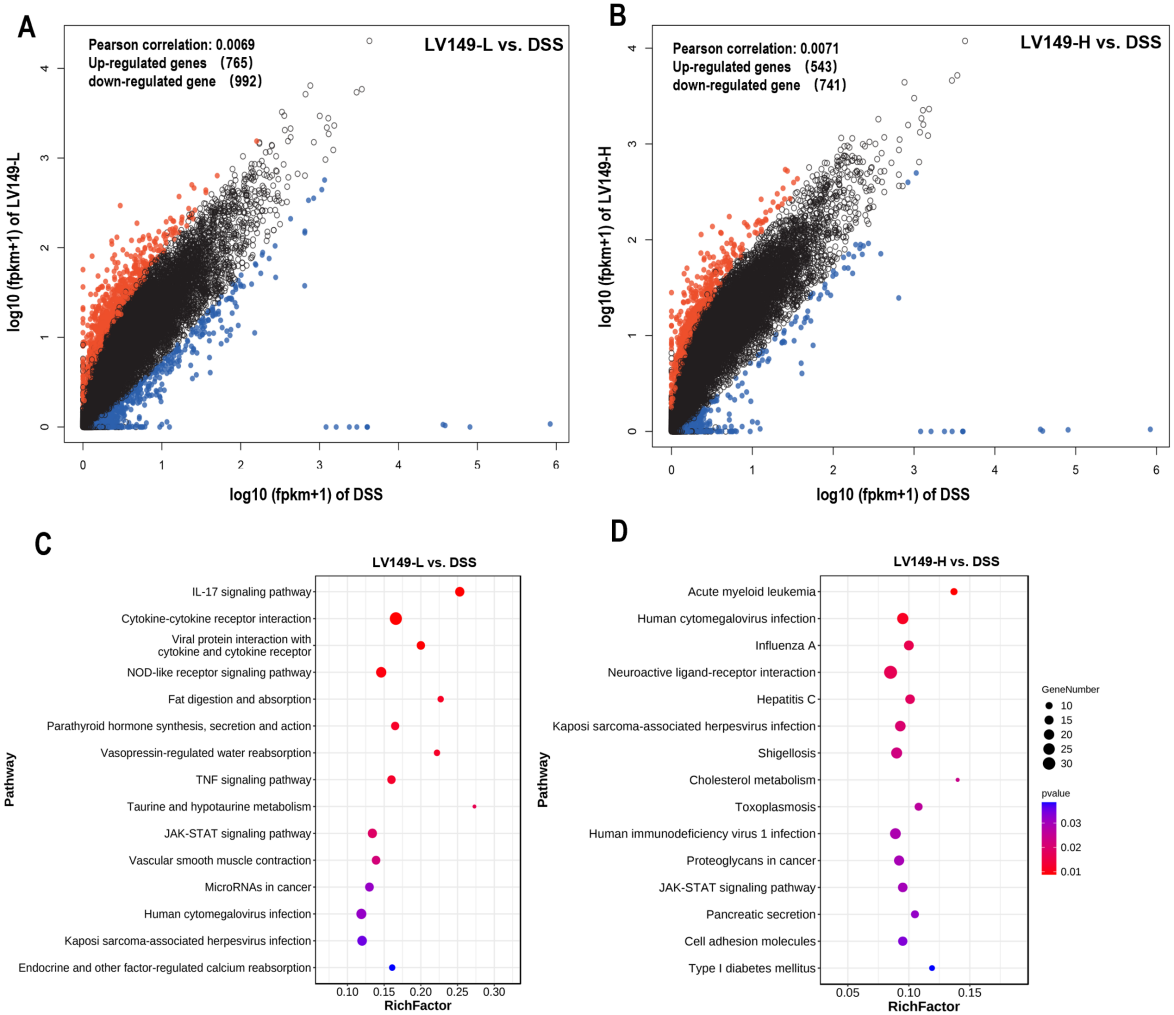
Transcriptome analysis showing LV149 alleviates DSS-induced colonic damage. **(A,B)** Scatter plots of differentially expressed genes (DEGs) comparing DSS with LV149-L and LV149-H groups (*n* = 4). **(C,D)** Top 15 signaling pathways enriched by DEGs, identified through KEGG analysis between DSS and LV149-L or LV149-H groups (*n* = 4).

Quality metrics of the filtered data showed that the Q30 base distribution ranged from 95.54 to 97.13%, with an average GC content of 46.44% (Supplementary Table S2). Reads were aligned to the reference genome, resulting in an average mapped rate of 89.99% (Supplementary Table S3). PCA results demonstrated high transcriptomic similarity among samples within each group (Supplementary Figure S4). Comparison between the DSS and LV149-L groups identified 1,757 differentially expressed genes (DEGs), with 765 upregulated and 992 downregulated genes (Figure 8A). Similarly, analysis of the DSS versus LV149-H groups revealed 1,284 DEGs, including 543 upregulated and 741 downregulated genes (Figure 8B).

Gene Ontology (GO) analysis showed significant enrichment of both upregulated and downregulated DEGs in processes such as cellular processes, biological regulation, and developmental processes (Supplementary Figure S5). KEGG pathway enrichment analysis of DEGs between the LV149-L and DSS groups revealed the top 15 enriched pathways, with notable inclusion of the IL-17 signaling pathway, cytokine-cytokine receptor interaction, TNF, and JAK-STAT signaling pathways, among others (Figure 8C). For the LV149-H versus DSS comparison, key enriched pathways included the JAK-STAT signaling pathway, acute myeloid leukemia signaling, human cytomegalovirus infection, pancreatic secretion, and cell adhesion molecules (Figure 8D).

**Figure 9:**
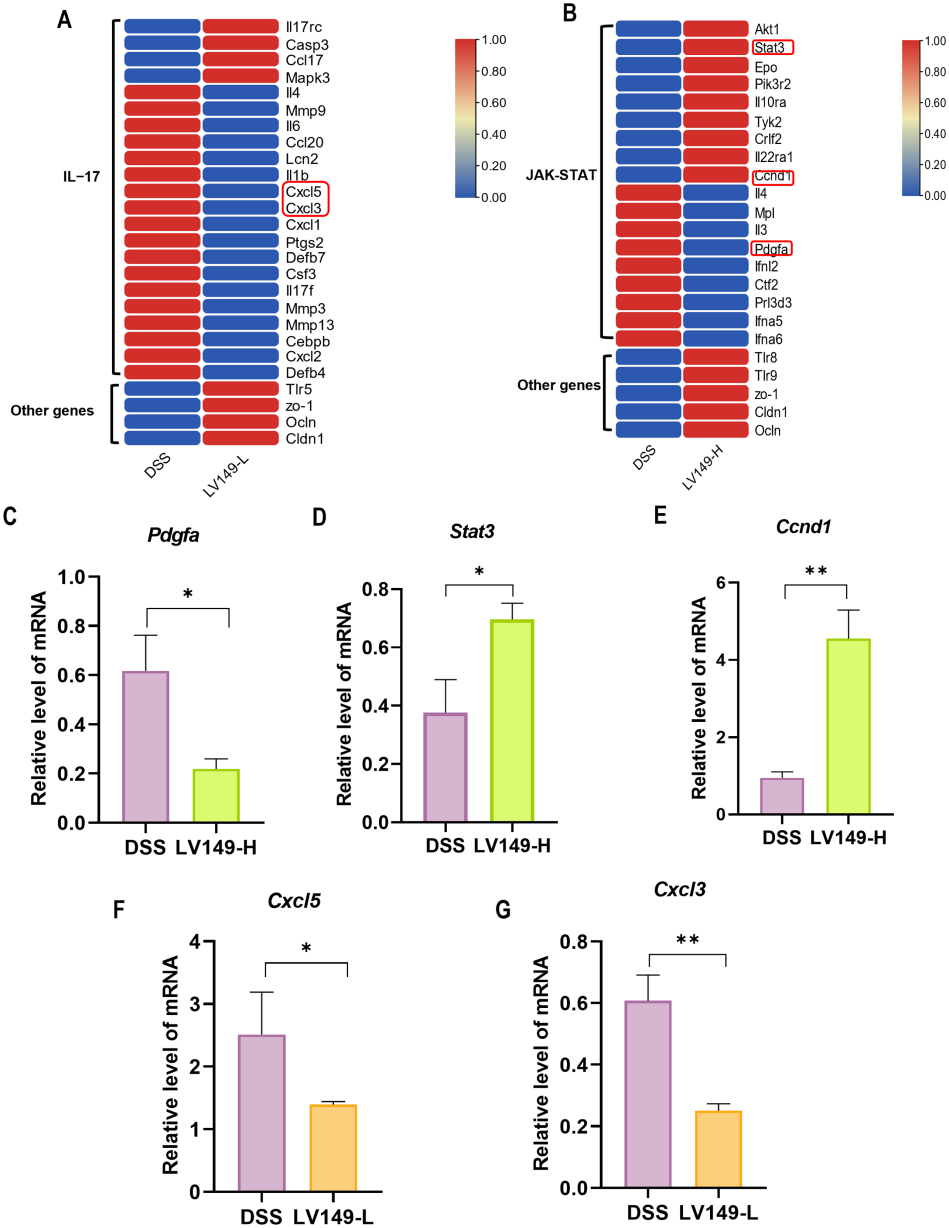
Heatmap of DEGs and validation of selected genes. **(A)** DEGs in the IL-17 signaling pathway. **(B)** DEGs in the JAK-STAT signaling pathway. **(C–E)** Validation of randomly selected genes *Pdgfa*, *Stat3*, and *Ccnd1* by qPCR (*n* = 3). **(F,G)** Validation of *Cxcl5* and *Cxcl3* by qPCR (*n* = 3). Data are presented as mean ± standard deviation. ^*^p < 0.05, ^**^p < 0.01, and ^***^p < 0.001 (unpaired *t*-test).

In the LV149-L vs. DSS analysis, expression patterns of DEGs related to transcription factors in the IL-17 signaling pathway were detailed in Figure 9A and Supplementary Table S4, where 22 DEGs were identified 4 upregulated and 18 downregulated. Key genes with increased expression included *Tlr5*, *ZO-1*, *Ocln*, and *Cldn1*. Similarly, Figure 9B and Supplementary Table S5 present DEGs associated with transcription factors in the JAK-STAT signaling pathway in the LV149-H vs. DSS analysis, where 18 DEGs were found 9 upregulated and 9 downregulated, with *Tlr8*, *Tlr9*, *ZO-1*, *Ocln*, and *Cldn1* showing higher expression.

Validation of the KEGG enrichment findings through qPCR analysis of *Cxcl3*, *Cxcl5*, *Stat3*, *Ccnd1*, and *Pdgfa* mRNA levels confirmed strong consistency with the transcriptomic data (Figures 9C–G).

### 3.6 Correlations between transcriptional and microbiota profiles in the LV149 and DSS groups

**Figure 10:**
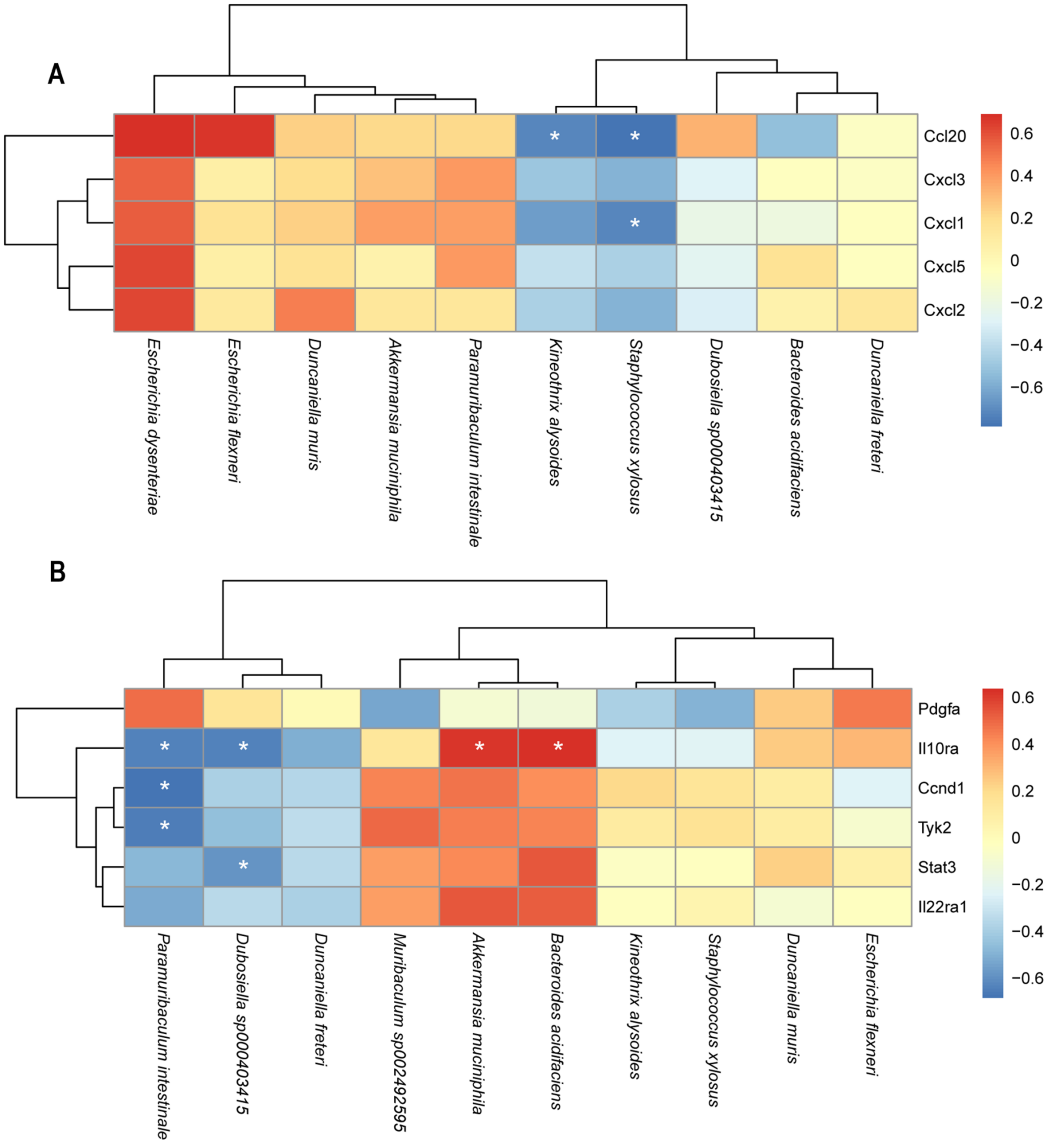
Heatmaps of the correlation analysis between DEGs in the transcriptome and gut microbiota at species level. **(A)** LV149-L vs. DSS **(B)** LV149-H vs. DSS. Red indicates a positive correlation a positive correlation, blue color indicates negative correlation. ^*^p < 0.05, ^**^p < 0.01, and ^***^p < 0.001 (Pearson’s correlation analysis).

**Figure 11:**
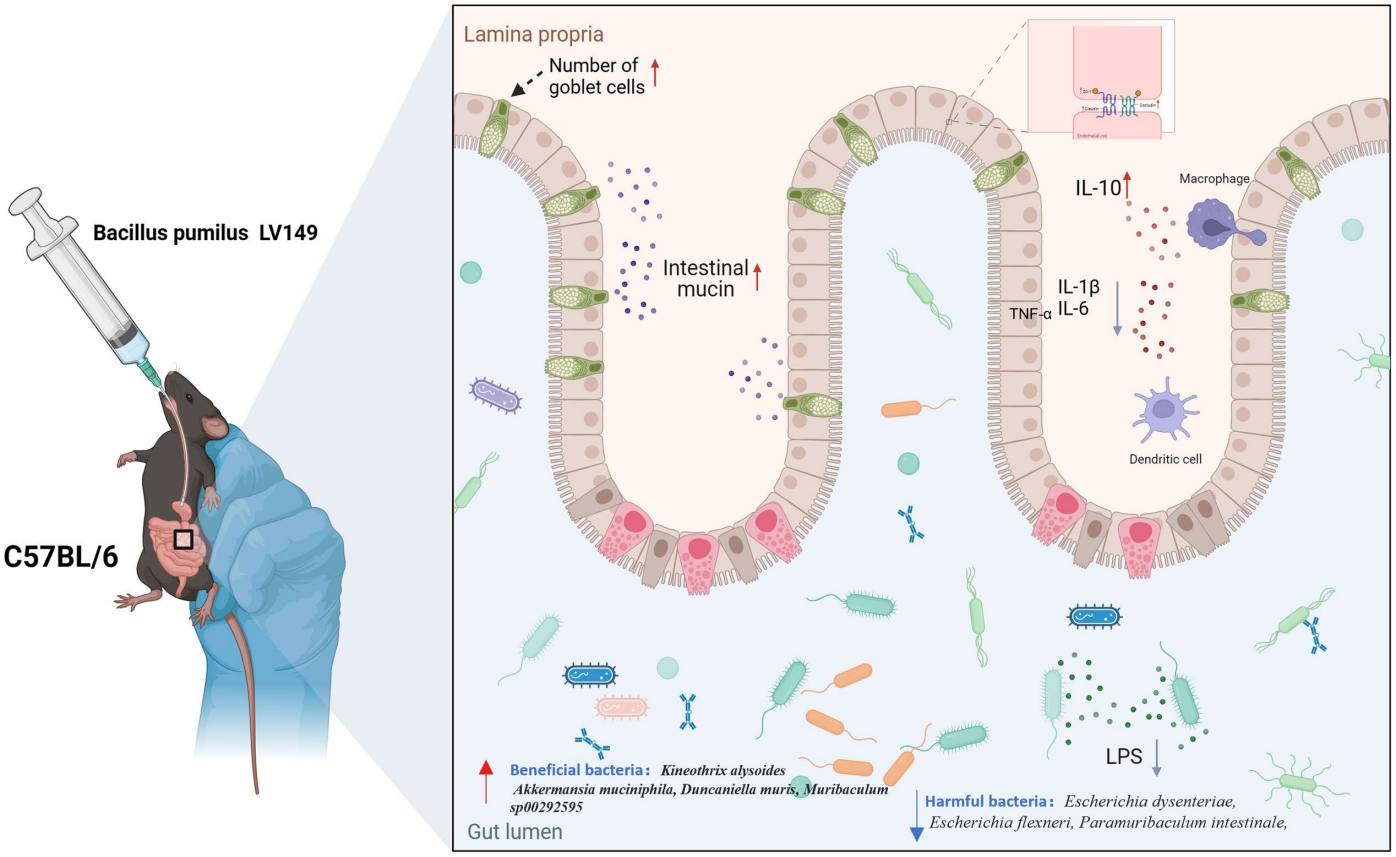
Diagram illustrating the reparative effects of LV149 on ulcerative colitis.

To further investigate the relationship between changes in gut microbiota and the expression levels of DEGs, a Pearson correlation analysis was conducted between the top 10 bacterial species and DEGs between the LV149 and DSS groups (Figures 10A,B). The analysis revealed that *Ccl20* was negatively correlated with *Staphylococcus xylosus* and *Kineothrix alysoides*. *Cxcl2* showed a negative correlation with *Staphylococcus xylosus*. Il10ra was negatively correlated with *Paramuribaculum intestinale* and *Dubosiella sp000403415*, while positively correlated with *Bacteroides acidifaciens* and *Akkermansia muciniphila*. Both *Ccnd1* and *Tyk2* were negatively correlated with *Paramuribaculum intestinale*. Additionally, *Stat3* was negatively correlated with *Dubosiella sp000403415* (see Figure 11).

## 4 Discussion

*Bacillus pumilus* LV149, a probiotic used in aquaculture to control Vibrio infections and maintain gut health (CN201210029204.9), has not been extensively studied for its effects. In this study, we explored the reparative effects of LV149 on a DSS-induced UC model mouse. Our findings demonstrated that LV149 significantly improved DSS-induced weight loss, increased DAI and shortened colon length in a dose-dependent manner. Histological analysis revealed a reduction in inflammatory cell infiltration and better-preserved crypt structures, which closely resembled those in healthy mice. These results suggest that LV149 has the potential to repair colon damage in the DSS-induced UC model mice, consistent with the protective effects of probiotics reported in other studies (Dong et al., 2022; Qu S. et al., 2021; Wang et al., 2022a).

The intestinal barrier, comprising the mucus layer, epithelial cells, tight junction proteins, and gut microbiota, plays a crucial role in maintaining gut integrity and protecting against harmful substances and pathogens (Baumgart and Dignass, 2002; Camilleri et al., 2012). In UC, disruption of this barrier allows intestinal antigens and pathogens to penetrate the mucosal layer, triggering inflammation and contributing to disease progression (Yu, 2018). Mucins, secreted by goblet cells, are essential for maintaining this barrier, and their loss is associated with severe intestinal dysfunction (Bergstrom et al., 2010). In our study, LV149 significantly increased the number of goblet cells and mucins in the colon tissues of DSS-induced UC model mice, suggesting that LV149 helps restore the mucus barrier.

Tight junction proteins, such as ZO-1, occludin, and claudin-1, are key components of the intestinal epithelial barrier, regulating epithelial permeability and preventing microbial translocation (Suzuki, 2020). In the DSS-induced UC model mice, tight junction proteins are typically downregulated, contributing to increased intestinal permeability. However, LV149 significantly upregulated the expression of these proteins in colon tissues, highlighting its potential to restore the integrity of the intestinal epithelial barrier and enhance gut health by preserving tight junction function.

In the progression of UC, inflammatory mediators (such as cytokines and chemokines) are abnormally activated. These mediators not only promote the persistence and expansion of inflammation but also further damage the colon tissue (Neurath, 2024). Therefore, improving the dysregulation of colonic inflammatory factors may be an effective strategy for repairing UC. Inflammatory cytokines such as IL-1β, IL-6, and TNF-α are key mediators of UC pathology. In DSS-induced UC, these cytokines are abnormally upregulated, promoting inflammation and further tissue damage (Dai et al., 2023; Li W. et al., 2022; Zhao et al., 2021). In our study, LV149 significantly reduced the expression of these pro-inflammatory cytokines, particularly at the higher dosage (LV149-H), while the lower dosage (LV149-L) showed a trend toward improvement. This dose-dependent effect suggests that LV149 modulates the inflammatory response by reducing the production of key inflammatory cytokines.

In addition, the anti-inflammatory cytokine IL-10 plays a crucial role in maintaining immune homeostasis in the gut. Jia et al. found that *Lactobacillus johnsonii* can release IL-10 through the TLR1/2-STAT3 pathway to alleviate UC (Jia et al., 2022). Yang et al. (2022) found that GPR120 can inhibit the development of colitis by regulating the production of IL-10 in intestinal CD4^+^ T cells. Our study showed that LV149 significantly increased IL-10 expression in a dose-dependent manner, suggesting that LV149 helps restore the balance of inflammatory mediators and promote intestinal healing.

LPS is a major component of Gram-negative bacterial membranes. LPS acts as a potent inflammatory stimulus, triggering the production of pro-inflammatory mediators such as cyclooxygenase-2 (COX-2) and prostaglandin E2 (PGE2), which are closely linked to IBD progression (Cianciulli et al., 2012). Previous studies have found that DSS-induced UC mice typically exhibit elevated levels of serum LPS (Gäbele et al., 2011; Qu Y. et al., 2021; Wang et al., 2022a). In this study, DSS administration led to an increase in serum LPS levels, further exacerbating the inflammatory response. Importantly, LV149 significantly reduced serum LPS levels in a dose-dependent manner, indicating that LV149 may help restore the intestinal barrier by preventing LPS translocation into the bloodstream. This reduction in LPS likely contributes to the observed alleviation of systemic inflammation and intestinal damage.

The gut microbiota is essential in inducing and regulating both local and systemic immune responses (Riazi-Rad et al., 2021). Inflammatory bowel disease is closely related to changes in gut microbiota composition (Ni et al., 2017). In patients with intestinal inflammation, there is a significant decrease in both the diversity and abundance of gut microbiota (Ott et al., 2004). Furthermore, compared to healthy mice, the gut microbiota in UC mice typically exhibit lower diversity and significant changes in microbial composition (Hao et al., 2021; Lu et al., 2022; Wang et al., 2022b). Subsequently, we further investigated whether LV149 would lead to changes in the structure of the gut microbiota, in order to understand the underlying reasons for the repair of intestinal damage in UC mice from different perspectives. In this study, DSS group mice displayed significant dysbiosis, characterized by reduced microbial diversity and increase in the relative abundance of harmful bacteria, including pro-inflammatory bacteria, *Proteobacteria*, *Deferribacteres*, and *Bacteroidetes*. Meanwhile, there was a reduction in the relative abundance of beneficial bacteria, particularly *Firmicutes*. In contrast to previous studies (Wang et al., 2022b), our research did not reverse the increase in *Bacteroidetes* abundance. We speculate that this may be attributed to differences in experimental conditions or strain specificity. However, further experiments are required to investigate the specific reasons behind this observation. Compared to mice in the DSS group, both doses of LV149 intervention reduced the relative abundance of harmful bacteria *Proteobacteria* and *Deferribacteres*, while only LV149-L increased the relative abundance of *Firmicutes*. We hypothesize that this phenomenon is closely related to the dosing concentration of LV149.

At the species level, we analyzed the changes in the relative abundance of the top 10 bacterial species to gain a more precise understanding of the shifts in the bacterial community. We found that compared to the DSS group mice, LV149-L significantly increased the relative abundance of the beneficial bacterium *Kineothrix alysoides*, which produces butyric acid (Choi et al., 2023; Haas and Blanchard, 2017), as well as *Duncaniella muris*, making their relative abundances more similar to those in the Ctrl group. In contrast to LV149-L, LV149-H not only increased the abundance of these two bacterial species but also significantly increased the relative abundance of the beneficial bacterium *Akkermansia muciniphila*, which is a symbiotic microorganism in the human gut that not only participates in host immune regulation but also enhances the integrity of intestinal epithelial cells and the thickness of the mucus layer, thereby promoting gut health (Zhang et al., 2019) and *Muribaculum sp002492595*. Meanwhile, compared to the DSS group mice, both doses of LV149 significantly reduced the abundance of the harmful genus *Escherichia*, which is an opportunistic pathogen involved in various intestinal and extra-intestinal infections (Denamur et al., 2021). Specifically, this may be due to the different doses of the medication, which regulate different species of *Escherichia*. LV149-L significantly reduced the relative abundance of *Escherichia dysenteriae*, whereas LV149-H significantly reduced the relative abundance of *Escherichia flexneri*. Additionally, both doses of LV149 significantly decreased the relative abundance of the gut symbiont *Paramuribaculum intestinale*, bringing its level closer to that of the normal group.

At the functional level, KEGG analysis revealed that the gut microbiota in the LV149-L group showed significant enrichment in immune-related and transcriptional pathways, which likely contribute to the modulation of inflammation and improvement of gut health. Conversely, the LV149-H group exhibited no significant enrichment in the infectious disease: bacterial category, suggesting that LV149 may also enhance gut health by improving microbiome functionality and reducing infection susceptibility. These findings provide further insight into how LV149 promotes intestinal repair.

Finally, transcriptomic analysis revealed that LV149 significantly upregulated the expression of tight junction-related genes (ZO-1, occludin, and claudin-1) in both LV149-L and LV149-H groups, supporting the histological findings of improved intestinal barrier function. Additionally, the DEGs between the LV149-L vs. DSS and LV149-H vs. DSS groups were significantly enriched in the JAK-STAT pathway associated with UC. However, Changes in DEGs from the LV149-L group did not show a positive effect on UC recovery via JAK-STAT regulation. This suggested that the mechanism by which LV149-L promotes UC recovery may differ from that of LV149-H. Further analysis revealed that DEGs in the IL-17 signaling pathway showed positive changes following LV149-L intervention, indicating that LV149-L may facilitate recovery through modulation of the IL-17 signaling pathway. Thus, we hypothesized that different dosages of LV149 may repair UC through distinct mechanisms, with LV149-L potentially acting via the IL-17 pathway and LV149-H via the JAK-STAT signaling pathway.

In the comparison between the LV149-L group and the DSS group, we found a large number of differentially expressed genes enriched in the IL-17 signaling pathway closely related to the immune system. Various chemokines (small proteins that guide white blood cells to sites of infection, injury, or inflammation) in this pathway, such as Cxcl1, Cxcl2, Cxcl3, and Cxcl5 and Ccl20 (Meitei et al., 2021; Mohammadi and Kariminik, 2021), showed significant differences in expression levels between the LV149-L and the DSS group.

In the comparison between the LV149-H group and the DSS group, we found that some differentially expressed genes associated with the improve of UC are enriched in the JAK-STAT signaling pathway. The pathway activated by IL-22R/IL-10R, utilizes STAT3 to promote the regeneration of intestinal stem cells and the repair of intestinal epithelial cells, increases the expression of mucins (Mizoguchi et al., 2018). Our transcriptomic analysis showed that in the LV149-H group, the upstream gene Tyk2 and pathway-related genes IL-22ra1, IL-10ra, and Stat3 are all significantly upregulated. Moreover, we also found several important upregulated genes within this pathway, including platelet-derived growth factor (PDGF) (Kumagai et al., 2001), Cyclin D1 (CCND1) (Baldin et al., 1993), and Toll-like receptors (TLRs) (Rakoff-Nahoum et al., 2004). It also suggests that LV149-H may enhance intestinal epithelial cell regeneration and mucosal barrier function through the modulation of the JAK-STAT pathway, thereby contributing to the recovery of intestinal pathological indicators. There are limitations in our study, a correlation between microbial signatures and gene expression in UC, yet lacks direct evidence linking these associations. Meanwhile, further studies are needed to explore the safety and the precise mechanisms underlying LV149’s effects on UC.

## 5 Conclusion

LV149 demonstrated significant potential in repairing the intestinal barrier, modulating inflammatory responses, and restoring gut microbiota balance in a DSS-induced UC model mice. These findings suggested that LV149 may offer a novel therapeutic strategy for UC, promoting intestinal health and potentially providing a valuable treatment option for UC patients.

## Data Availability

The datasets presented in this study can be found in NCBI with accession number: PRJNA1142690 and PRJNA1145126.

## Footnotes

^1^ http://rdp.cme.msu.edu/

^2^ http://doi.org/10.1016/j.taap.2020.114900

^3^ https://ccb.jhu.edu/software/hisat2/index.shtml

^4^ https://github.com/tanghaibao/GOatools

^5^ http://kobas.cbi.pku.edu.cn/home.do

## Glossary

IBD: Inflammatory bowel disease
UC: Ulcerative colitis
DAI: Disease activity index
DSS: Dextran sulfate sodium
H&E: Hematoxylin and eosin
IF: Immunofluorescence
IL: Interleukin
LDA: Linear discriminant analysis
LEfSe: LDA coupled with effect size
LPS: Lipopolysaccharides
OTUs: Operational taxonomic units
PCoA: Principal coordinate analysis
PCA: Principal component analysis
TJs: Tight junctions
TNF-α: Tumor necrosis factor-α
Il4: Interleukin 4
Mmp9: Matrix metallopeptidase 9
Il6: Interleukin 6
Ccl20: Chemokine (C-C motif) ligand 20
Lcn2: Lipocalin 2
Il1b: Interleukin 1 beta
Cxcl5: Chemokine (C-X-C motif) ligand 5
Cxcl3: Chemokine (C-X-C motif) ligand 3
Cxcl1: Chemokine (C-X-C motif) ligand 1
Ptgs2: Prostaglandin-endoperoxide synthase 2
Defb7: Defensin beta 7
Csf3: Colony stimulating factor 3 (granulocyte)
Il17f: Interleukin 17F
Mmp3: Matrix metallopeptidase 3
Mmp13: Matrix metallopeptidase 13
Cebpb: CCAAT/enhancer binding protein (C/EBP), beta
Cxcl2: Chemokine (C-X-C motif) ligand 2
Defb4: Defensin beta 4
Il17rc: Interleukin 17 receptor C
Casp3: Caspase 3
Ccl17: Chemokine (C-C motif) ligand 17
Mapk3: Mitogen-activated protein kinase 3
Tlr5: Toll-like receptor 5
ZO-1: Zonula occludens-1
Ocln: Occludin
Cldn1: Claudin 1
Akt1: Thymoma viral proto-oncogene 1
Stat3: Signal transducer and activator of transcription 3
Epo: Erythropoietin
Pik3r2: Phosphoinositide-3-kinase regulatory subunit 2
Il10ra: Interleukin 10 receptor, alpha
Tyk2: Tyrosine kinase 2
Crlf2: Cytokine receptor-like factor 2
Il22ra1: Interleukin 22 receptor, alpha 1
Ccnd1: Cyclin D1
Mpl: Myeloproliferative leukemia virus oncogene
Il3: Interleukin 3
Pdgfa: Platelet derived growth factor, alpha
Ifnl2: Interferon lambda 2
Ctf2: Cardiotrophin 2
Prl3d3: Prolactin family 3, subfamily d, member 3
Ifna5: Interferon alpha 5
Ifna6: Interferon alpha 6
Tlr8: Toll-like receptor 8
Tlr9: Toll-like receptor 9
