# Supplementary figures and images for: Probiotic Bacillus pumilus LV149 enhances gut repair, modulates microbiota, and alters transcriptome in DSS-induced colitis mice
Source: Front Microbiol. 2025 Jan 7;15:1507979. doi: 10.3389/fmicb.2024.1507979 (PMC11753000; doi:10.3389/fmicb.2024.1507979)

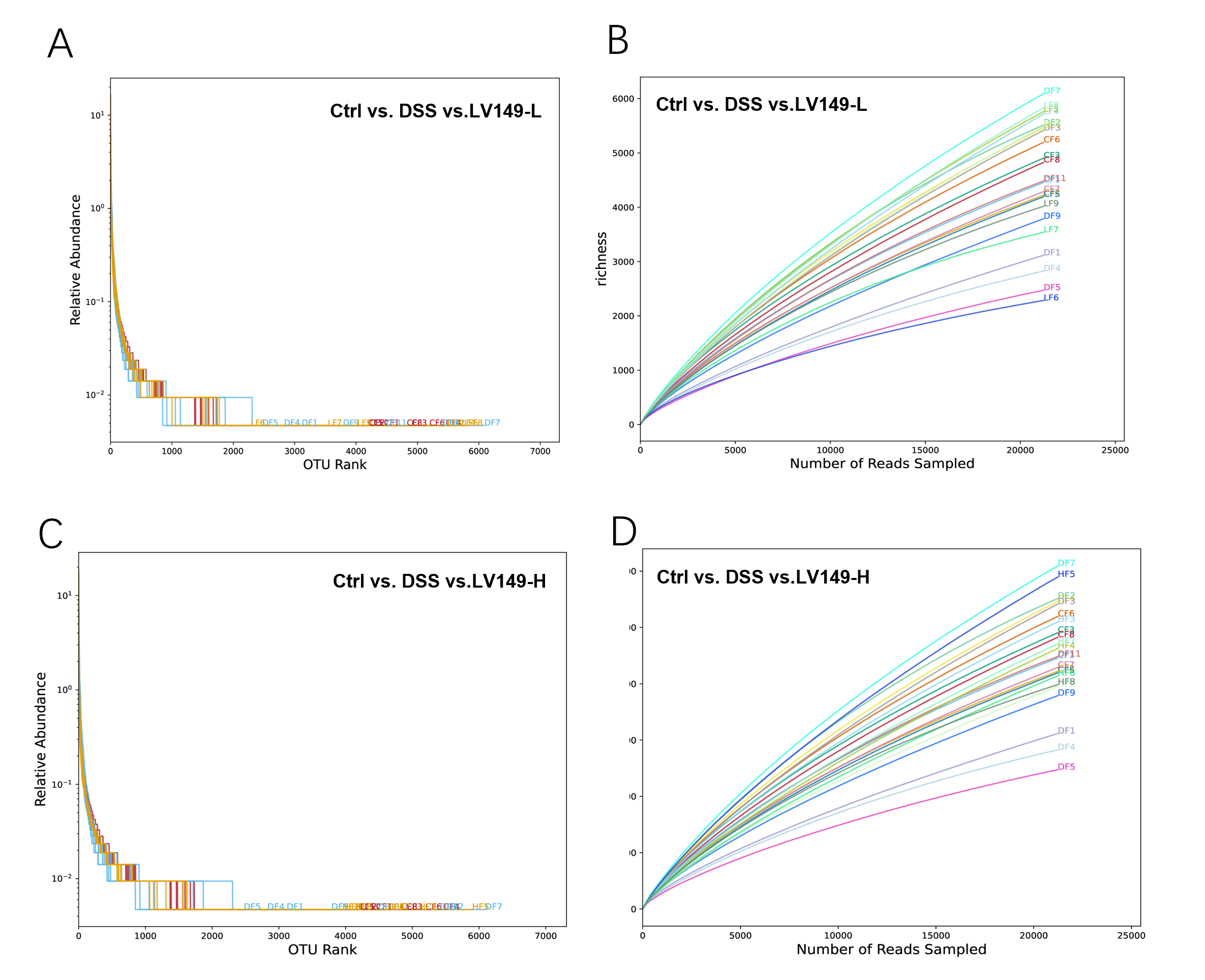

Supplement: Supplementary file 6 [file Image_1.TIF]

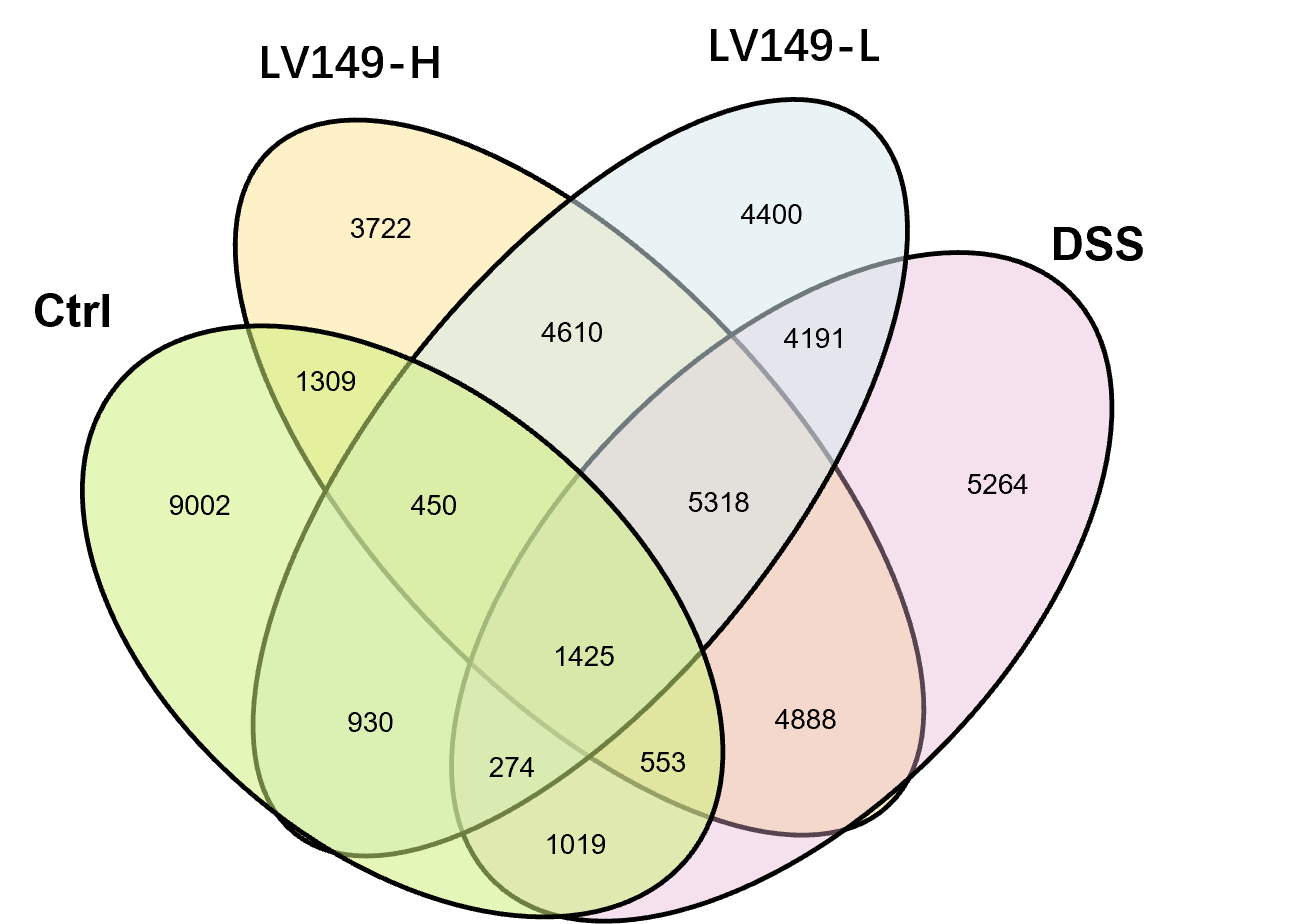

Supplement: Supplementary file 7 [file Image_2.TIF]

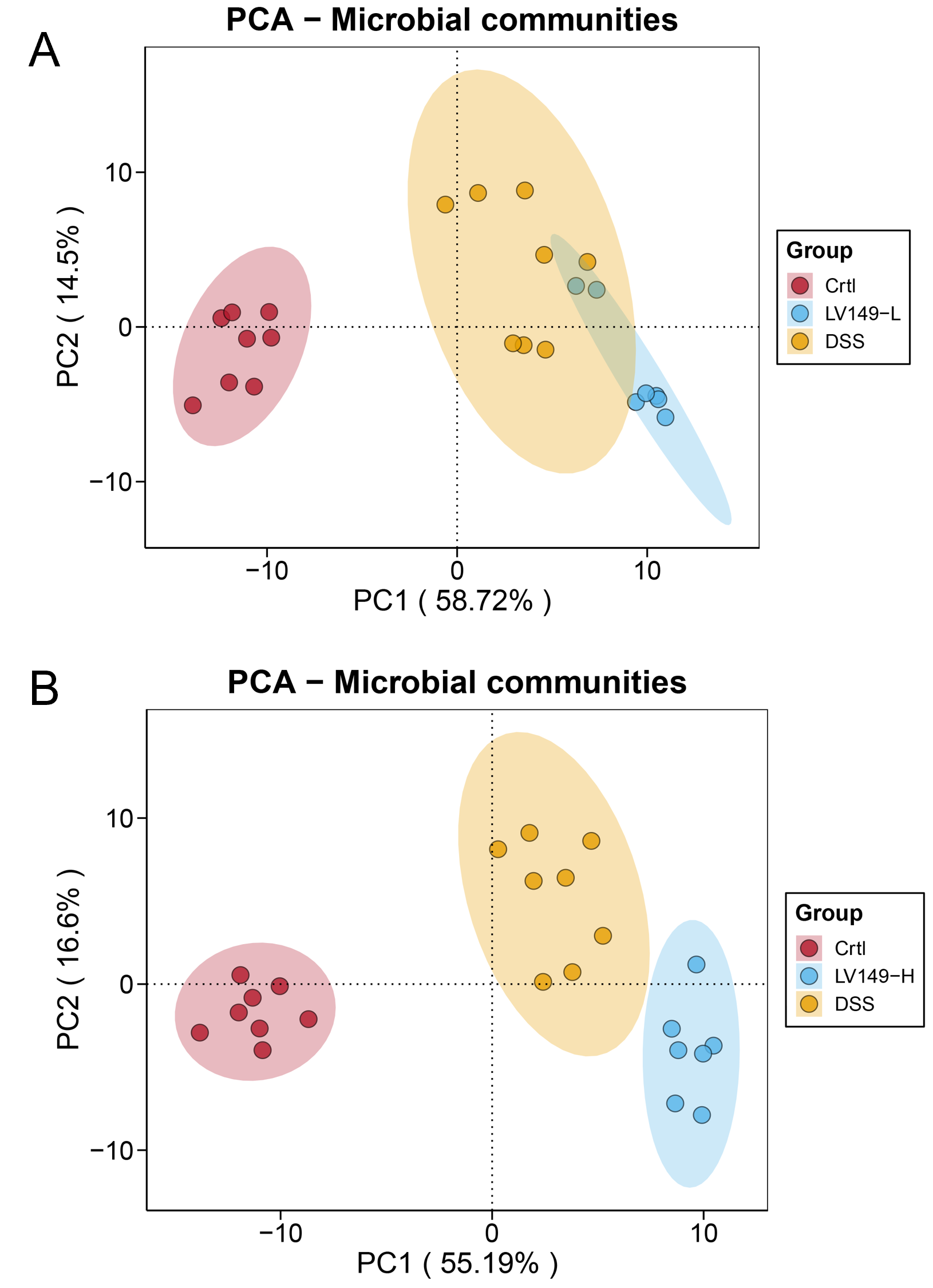

Supplement: Supplementary file 8 [file Image_3.TIF]

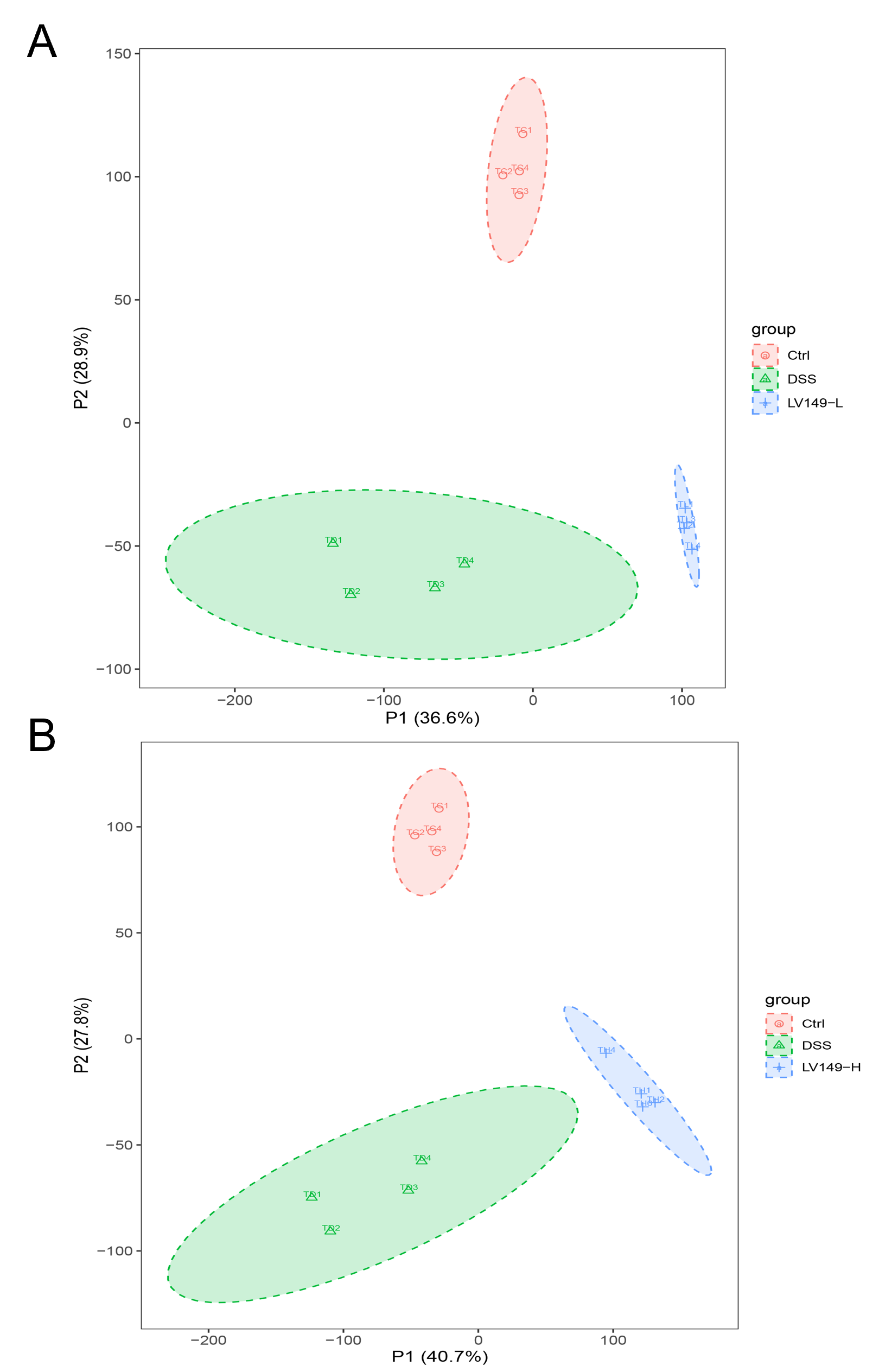

Supplement: Supplementary file 9 [file Image_4.TIF]

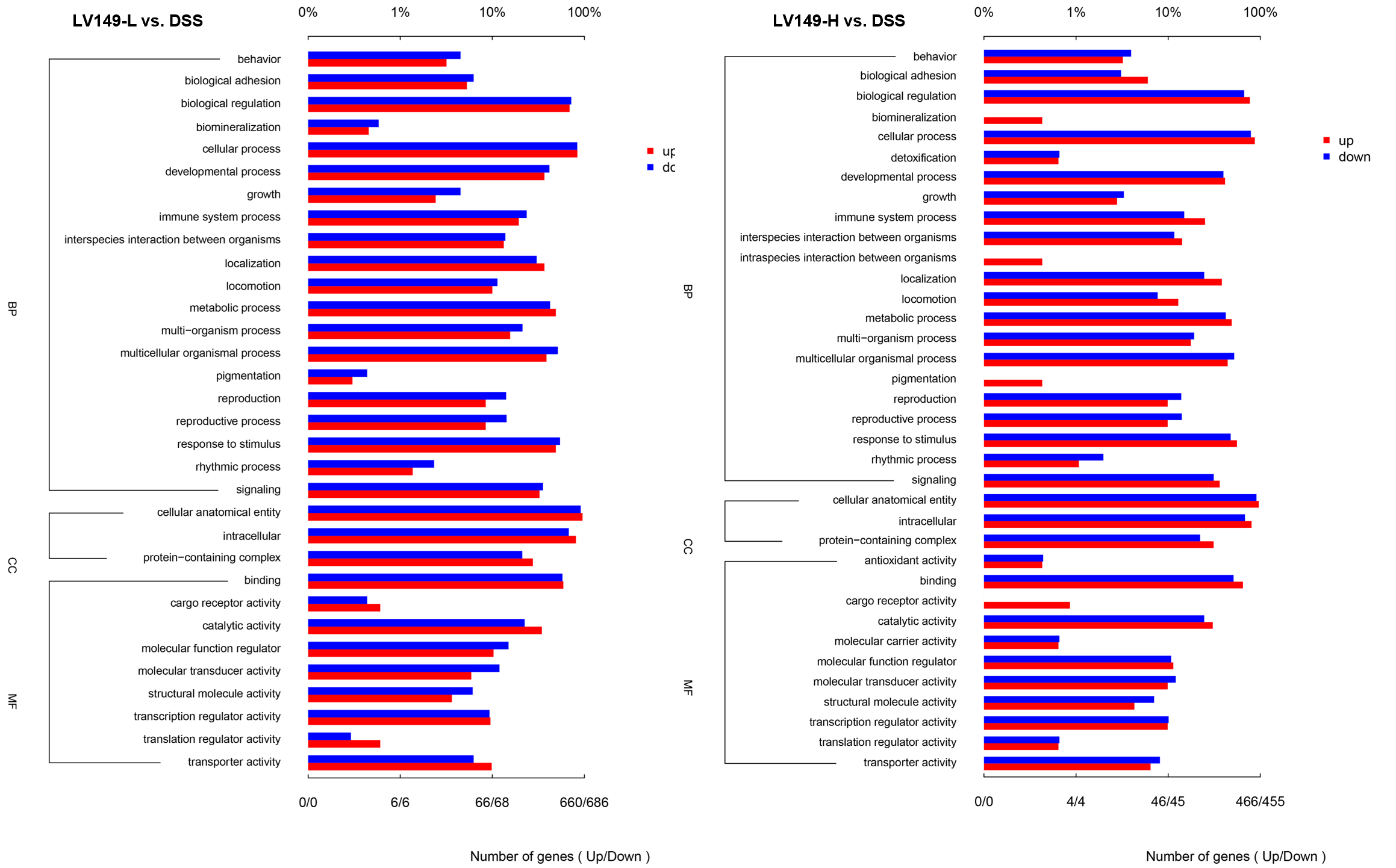

Supplement: Supplementary file 10 [file Image_5.TIF]
